# Supplementary material for: Are carbon emissions trading and green financial instruments synergistic? -Comprehensive quantitative research based on content analysis
Source: PLoS One. 2024 Mar 7;19(3):e0298601. doi: 10.1371/journal.pone.0298601 (PMC10919601; doi:10.1371/journal.pone.0298601)
Supplement: S1 Data — (ZIP) [file pone.0298601.s001.zip › Data/地级市Z_F工作报告.zip!/╕╩╦α/┬ñ─╧╩╨/└┤╫╘╣1⁄2╓┌║┼í╛┬φ┐╦╩2╛▌═°í┐_2015.docx]

各位代表：

　　现在，我代表市人民政府向大会作工作报告，请予审议，并请各位政协委员和列席人员提出意见。

　　2014年工作回顾

　　2014年，面对复杂多变的经济形势和艰巨繁重的改革发展任务，我们在市委的坚强领导下，全面贯彻党的十八届三中、四中全会精神，认真落实省委、省政府重大决策部署和市委全委扩大会、市三届四次人代会精神，坚持稳中求进总基调，全力落实“433”发展战略，统筹推进稳增长、调结构、促改革、惠民生各项工作，较好地完成了全年目标任务。全市主要经济指标增速高于全省平均水平。全市完成生产总值262.5亿元，同比增长9%；其中一、二、三产增加值同比分别增长6.2%、10.5%、9.2%；完成固定资产投资532.9亿元，增长21.4%；完成大口径财政收入49.3亿元、公共财政预算收入23.9亿元，分别增长18.2%和25.2%；完成 农 民 人 均 纯 收 入4023.7元 ， 增 长13.8%；完成城镇居民人均可支配收入17001.3元，增长9.3%；完成社会消费品零售总额72.3亿元，增长12.7%；完成外贸进出口总额2171万美元，增长102%；全市新增城镇就业3.1万人，城镇登记失业率2.26%；居民消费价格总水平涨幅2.4%。其中，农民人均纯收入、社会消费品零售总额和一产增加值的增幅分别排在全省第1、第2和第3位。

　　一、经济转型升级步伐加快，产业培育迈上新台阶

　　大力推进调结构、稳增长、促转型，着力破解发展难题，经济保持了健康发展、平稳运行的良好态势。现代农业发展步伐进一步加快。认真落实强农惠农政策，创新农业发展机制，特色农业标准化、合作化、市场化程度不断提高，规模化经营取得了好成绩。粮食总产实现八连增，达到112万吨。花椒、核桃、油橄榄等经济林果产量、产值同比大幅增长，苗木产业、畜牧水产、规模化养殖比重进一步提升。完成核桃高接换优486万株。农民合作社建设步伐加快，“十到社”工作初见成效。土地确权登记颁证试点任务全面完成，土地、林地流转面积进一步扩大。农产品质量安全监管走在全省前列。成功举办了各类特色农产品产销对接洽谈会，在北京举办了油橄榄产业新闻发布会。我市被国家林业局确定为全国油橄榄示范基地。工业经济实现较快发展。礼县紫金矿业、文县万利铁合金公司等重点企业达产达标，成县祁连山水泥技改扩能、苗家坝电站等一批重大项目建成投产，新增规模以上工业企业7户。工业用电量增幅全省第一。白酒、建材、非金属、制药等产业产能不断提升。黄金产业发展势头强劲，全年产量达到7吨以上。 郭家沟铅锌矿按照世界领先、国内一流绿色理念加快建设。开发区基础设施、土地储备、企业入园取得实质性进展。全年完成规模以上工业增加值40.1亿元，同比增长10%，增速位居全省前列。节能节水工作稳步推进。非公经济发展迈出新步伐。文化旅游产业发展步伐加快。官鹅沟国家森林公园和礼县大堡子山国家考古遗址公园等重点项目启动实施。新增4A级景区3个，总数达到7个，位列全省第二，我市75个村被列入全国乡村旅游扶贫重点村。成功举办第六届乞巧女儿节与妇女发展国际论坛等重大活动。文化产业项目招商取得新进展。文化资源保护工作迈上新台阶，古建筑、古民居调查摸底测绘工作顺利完成，乞巧节申报世界非物质文化遗产工作积极推进。主要景区实现无线网络全覆盖，智慧旅游取得初步成效。全市完成文化产业增加值4.77亿元，增长24.5%。电子商务实现突破性发展。淘宝网“特色中国·陇南馆”开馆运营，全市开办网店5923家，实现销售总额7.71亿元，开辟了农民增收、农业增效和农村发展的新途径，闯出了一个贫困地区发展农产品电子商务的“陇南模式”。国务院扶贫办确定我市为全国电商扶贫试点市。现代服务业和物流园区发展进一步加快。

　　二、项目建设取得新进展，经济社会发展条件持续改善

　　坚持领导干部包抓重点项目，1145个“3341”项目工程加快推进，完成投资580.7亿元。成武高速建成通车，成为连接北部六县和南部三县区的黄金大动脉，全市高速公路总里程达到220公里。十天高速、渭武高速试验段、成州机场建设进展顺利。武九高速、徽两高速和天平铁路南延线前期工作有序推进。国省干线联网路、国道二级公路改造顺利实施。市区长江大道东西延伸一期工程建成通车。硬化通村公路3000公里。自筹资金建成了武都五马至康县阳坝公路，为打造甘陕川旅游“金三角”生态腹地、推动沿线群众脱贫致富创造了条件。民生水利项目全面推进，解决了25万农村人口饮水问题。电网建设进一步加快，2个110千伏变电站顺利投运，成县农网改造实现全覆盖。信息化建设得到加强。积极融入长江经济带和成渝经济圈，对外交流、院地院企合作取得新进展，向南开放迈出实质性步伐，招商引资到位资金同比大幅增长。

　　三、特困片区扶贫攻坚加快推进，精准扶贫取得实质性进展

　　坚持真扶贫、扶真贫，深入落实“1236”扶贫攻坚行动，启动实施25个特困片区扶贫攻坚工程，实现了扶贫理念和扶贫方式的重大转变。实行市县区领导包抓特困片区责任制，坚持资金向片区倾斜、项目向片区整合，形成了合力攻坚的良好格局。整合行业部门项目资金31亿元，争取财政专项扶贫资金4.3亿元，其中93%的专项资金安排在特困片区。120个整村推进项目全面完成。建档立卡工作扎实开展，为精准扶贫奠定了基础。油橄榄、中药材国家级产业化扶贫试点项目申报工作积极推进。劳务输转力度进一步加大，“两后生”培训和职业技能培训、鉴定工作成效显著，“双五千”人才输送取得新进展。易地扶贫搬迁顺利完成。“双联”行动深入推进，建立了驻村帮扶工作队，实现了对所有贫困村的全覆盖联系。建设生态文明新农村315个。全市预计减少贫困人口20万人。

　　四、城镇带动效应逐步显现，城乡一体化建设取得新成效

　　市区、八县县城和建制镇规划编制工作全面完成，城镇建设协调推进。市区东江新区建设明显加快，滨江公园等重点项目全面建成，陇南火车站、阶州文化城等项目快速推进，钟楼滩、吉石坝等新区建设进一步加快。各县县城旧城改造、路网建设、供水供热、垃圾污水处理等重点市政项目取得新进展。数字城市建设和智慧城市试点工作稳步推进，城市管理运营创新升级。新型城镇化试点工作取得新进展，礼县、康县被列为全省新型城镇化试点县，成县小川镇、宕昌哈达铺镇被列为全省新型城镇化试点镇。在全省率先启动小城镇综合改革试点工作，编制完成全市9个试点镇修建性详规。设立小城镇综合改革奖补资金1亿元，试点镇重点项目建设进展良好，各项综合配套改革同步推进。全市城镇化率预计达到27%以上。美丽乡村建设取得明显成效，全市新建省级美丽乡村17个。全省改善农村人居环境行动现场会在康县召开，康县美丽乡村建设模式和成功经验得到充分肯定，并在全省大力推广。传统古村落保护项目申报工作取得积极进展。

　　五、环境保护和治理得到加强，生态文明建设取得新进展

　　生态治理、环境保护和林业生态建设进一步加快，“两江一水”治理规划、水生态文明城市建设试点顺利推进。争取到新一轮退耕还林17万亩，占全省65万亩的26.2%。植树造林工作成效明显。江河干支流、中小河流和山洪沟道治理进展良好，水土保持工程取得新成效。污染减排和重金属污染治理扎实开展，铅锌选矿企业污水治理项目、西和县重金属污染治理示范县项目取得较好进展，大气环境治理持续推进。矿山、尾矿库专项整治行动取得实效。主要污染物总量减排指标控制在省上下达的指标以内，农村环境整治进一步加强，农村清洁能源建设取得较好成效，全市没有发生重大环境事件。两当生态示范县创建工作全面展开。地质灾害防治成效显著，陇南山区国家级地质灾害防治管理及监测预警示范区建设顺利推进，水资源实时监控与管理系统进入调试验收阶段，自然灾害监测预警系统建设得到加强，新一代天气雷达监测工程进展顺利，防灾减灾能力进一步提升。

　　六、财政保障能力不断提升，金融支撑实现新突破

　　财政征收进一步加强，全市大口径财政收入和公共财政预算收入逆势增长，公共财政预算支出稳步增长，有力保障了项目建设、产业培育、民生工程等各类重点支出。金融服务保障能力明显增强，全市金融机构贷款余额为376.5亿元，同比增长22.8%，存贷比达到60.19%。涉农贷款、“三权”抵押贷款、双联惠农贷款实现较快增长。市县区融资平台和土地储备中心建设取得阶段性进展。普惠金融加快发展，启动农村物权抵押贷款交易试点工作，成立首家农村物权抵押贷款交易中心。中药材、易地搬迁、畜牧业、设施蔬菜、电子商务等信贷扶持政策得到落实。中小微企业融资“资金池”试点工作启动实施，34户企业在甘肃股权交易中心挂牌交易。落实银企对接签约资金7.4亿元。甘肃银行在我市9县区设立支行，兰州银行东江支行正在筹建当中。棚户区改造项目申报和贷款争取工作取得阶段性成效。

　　七、社会事业和民生工程协调推进，人民群众得到更多实惠

　　教育惠民政策全面落实，全年教育专项支出达到10.99亿元。中小学教育质量稳步提升，陇南市一中顺利通过省级示范性高中评估验收，成为我市第一所省级示范性高中。农村薄弱学校改造项目顺利推进。职业教育加快发展，学前教育基础设施建设进展良好。科技创新得到加强，科技特派员工作在助推双联行动中发挥了积极作用。农村文化基础设施建设进一步加快，公共文化服务水平有较大提升。基本医疗保障水平得到提高，农村卫生服务体系不断健全，公共卫生服务项目深入实施，居民健康素养提升工程稳步推进，中医药特色机构创建得到加强。7个体育惠民工程建成投用，全民健身网络体系不断完善，在省第十三届运动会上取得历史最好成绩。社会保障和救助工作进一步加强，全市社会保险“五险合一”工作稳步开展，被征地农民养老保险工作进展顺利，城乡居民社会养老保险补助标准进一步提高，农村低保动态管理机制运行良好，城乡低保、农村五保标准有新提升。各项就业扶持政策得到落实，超额完成省上下达的城镇新增就业任务，促进高校毕业生就业创业工作取得积极成效。农村危旧房改造、保障性住房建设和城市棚户区改造取得新进展。地震和暴洪灾害灾后重建项目进展顺利。省上确定的10项23件和市上确定的10件民生实事全部办结。各类民生支出达到135亿元。严格落实安全生产责任，安全监管进一步加强，全年没有发生较大事故，安全生产形势保持稳定。物价监管和食品药品安全监管措施全面落实。矛盾纠纷排查调处和信访工作深入开展，应急处置工作机制不断完善，社会服务管理创新有效推进，县区、乡镇基本实现社会服务管理中心全覆盖，90%以上的村建立了村级社会服务管理站，平安建设取得新成效，社会大局保持和谐稳定。民族宗教、外事侨务、统计、审计、档案、地方志、气象、“双拥”、人防、民兵预备役和妇女儿童、残疾人、老龄、慈善、红十字会等各项事业取得新进步。

　　八、重点领域改革深入推进，经济社会发展活力持续增强

　　积极推进行政审批制度改革，共取消、下放、合并行政审批事项122项。整合资源，新建了市区两级政务服务大厅。公共资源交易平台建设日趋完善，各类公共资源交易项目基本实现进场交易，交易工作更加规范透明。国有资产管理体制改革和部门预算改革有序推进。乡改镇工作取得新进展。工商、质监、卫生计生机构改革顺利完成。食品药品监管体制改革全面完成，监管能力明显提升。公立医院综合改革试点工作顺利实施，市县乡卫生资源整合取得阶段性进展。单独两孩政策稳妥有序实施。对陇南卫校、陇南师专继续教育中心和武都区职业中专三校资源进行整合，为创建陇南卫生职业技术学院打下了基础。

　　政府系统按照市委的安排部署，扎实开展了党的群众路线教育实践活动，作风建设取得新成效。全市“三公”经费开支比上年下降38.14%，文件数量减少13%，会议数量减少29%。

　　各位代表，过去一年的成绩来之不易。这些成绩的取得，是省委、省政府关怀支持的结果，是市委正确领导、科学决策的结果，是市人大、市政协和社会各界监督支持的结果，是广大干部职工和全市人民艰苦奋斗、顽强拼搏的结果。在这里，我代表市人民政府，向奋战在各条战线上的广大干部群众，向驻陇南人民解放军、武警官兵和中央、省驻陇南单位，向各民主党派、工商联、无党派爱国人士、离退休老干部，向奋战在陇南的重大建设项目施工人员，向所有关心支持陇南经济发展、社会进步的各界人士表示衷心的感谢和崇高的敬意!

　　在充分肯定成绩的同时，我们也清醒地认识到，经济社会发展还面临着许多矛盾和问题。主要表现在：固定资产投资渠道狭窄，新常态下的固定资产投资增长机制尚未建立，招商引资项目签约多、落地少，保持投资快速增长难度较大；结构性矛盾较为突出，新常态下调结构、转方式任务艰巨，工业产业结构仍然单一，现代服务业规模小、比重低；农业生产规模化、集约化程度不高，农业产业化经营水平仍然较低；基础设施仍然薄弱，高速公路密度小，乡村公路等级低，农村公共服务设施欠账较多；城乡居民收入增长缓慢，持续增收面临较大难度。面对上述困难和问题，我们必须保持清醒头脑，采取有效措施认真加以解决。

　　2015年主要工作任务

　　各位代表，2015年是全面深化改革的关键之年，是全面推进依法治市的开局之年，也是全面完成“十二五”规划的收官之年。今年政府工作的总体要求是：深入贯彻党的十八大和十八届三中、四中全会精神，按照中央、全省经济工作会议精神和省委、省政府重大决策部署，在市委的坚强领导下，坚持稳中求进工作总基调，坚持以提高经济发展质量和效益为中心，主动适应经济发展新常态，着力转方式、调结构，强基础、兴产业，稳增长、惠民生，持续推进改革创新，更加注重开放开发，推动“433”发展战略全面实施，力争全面完成“十二五”规划任务，促进经济平稳健康发展和社会和谐稳定。

　　经济社会发展的主要预期目标是：生产总值增长8.5%，固定资产投资增长18%，规模以上工业增加值增长10%，公共财政预算收入增长10%，城镇居民人均可支配收入增长10%，农民人均纯收入增长13%，社会消费品零售总额增长12%，城镇登记失业率控制在4%以内，居民消费价格涨幅控制在3%以内，万元地区生产总值能耗降幅和主要污染物排放指标完成省定目标。这些主要预期指标，是市委、市政府充分考虑经济发展新常态和陇南阶段性特征，综合平衡各种有利条件和不利因素后研究确定的，也是根据“433”发展战略，主动适应向南开放，为实现与全国全省同步进入小康社会奋斗目标来谋划的。

　　为了确保完成全年各项目标任务，重点抓好以下工作：

　　一、深入实施特困片区扶贫攻坚，推动农业和农村发展

　　坚持以农民持续增收为核心，以特困片区扶贫攻坚为主线，以全面深化农业农村改革为动力，着力推进农业农村经济快速发展。一是全面推进25个特困片区扶贫攻坚。把特困片区扶贫与精准扶贫相结合，按照规划到村、措施到户的要求，把90%以上的扶贫资金和80%以上的涉农资金投放到特困片区，把项目倾斜到特困片区，加快推进特困片区路、水、电、房等基础设施建设，整体改善特困片区基础设施条件。抓好整村推进项目。谋划实施好各项专项扶贫措施，打好扶贫攻坚“组合拳”。启动实施电商扶贫试点工程，加快培育电商扶贫产业，创建全国电商扶贫示范区。做好旅游扶贫示范县的争取和实施工作，特别是要抓好五阳路沿线旅游扶贫规划和示范村建设，在康县启动实施农村旅游“十村百户千床”工程，让生态旅游、新农村旅游、养生旅游真正成为农民增收致富的重要产业。鼓励社会资本和民营企业参与农业产业开发和扶贫开发。进一步加强劳动力技能培训和劳务输转，完成“两后生”职业技能学历教育培训9000人，开展劳动力技能培训3.5万人以上，争取输转劳务72万人。深化拓展“双联”行动，发挥好驻村帮扶工作队的作用，促进双联与扶贫深度融合。加大易地扶贫搬迁力度。新建生态文明新农村210个。全市减少贫困人口13.5万人。为有条件整体脱贫的县乡村奠定基础，做好准备。二是开展“特色产业提质增效年”活动。落实好特色产业国家扶持政策，引导适度规模经营。以经济林果、中药材、苗木、畜牧养殖等为重点，稳定基地面积，打造一批农业特色产业提质增效示范点。加快徽县泥阳等现代农业科技示范园区建设，抓好核桃高接换优和经济林综合管理，持续开展油橄榄引种和示范推广，加快礼县、西和苹果矮砧密植示范点建设，着力推进标准化规模养殖，大力发展林下经济和生态养殖，把陇南建设成全省生态鸡放养基地。大力推进“十到社”工作，加快建设100个市级示范社和150个电商示范社。加大农业发展基金对龙头企业扶持力度，加快培育油橄榄龙头企业、中药材加工仓储营销企业和农产品精深加工企业。办好核桃、花椒、油橄榄、苹果、苗木、中药材等特色农产品产销对接洽谈会。全面开展土地承包经营权确权登记颁证，推进农村土地经营权有序流转。抓好研发中心建设，加快推进标准化生产体系建设，推广中药材GAP标准化生产，抓好农产品质量安全监管，“三品一标”农产品生产面积和养殖比重达到50%以上。三是进一步抓好粮食生产。稳定粮食播种面积，加快推广全膜双垄沟播、测土配方施肥、粮油高产创建等科技增粮措施和高效农田节水技术，加强病虫害防治，落实好粮食直补、良种补贴等惠农政策，确保粮食总产稳定在100万吨以上。

　　二、加快工业经济发展，推动工业转型升级

　　加快培育有色冶金、非金属、白酒、建材、中医药制造、农特产品加工等主导产业，推动工业#from 本文来自学优网http://www.gkstk.com end#转型升级。一是加快重点工业项目建设。实施市列重点工业项目20个以上，抓好郭家沟铅锌矿采选、金徽酒业技术改造、恒丰农林商贸公司5万吨核桃露加工等项目建设，加快紫金矿业黄金冶炼厂、中宝矿业矿石采选等项目前期工作。积极谋划传统产业更新换代和循环利用项目。加大矿产资源勘查力度，积极开展风险探矿，增强工业发展后劲。二是推动重点企业发展。加强协调服务，促进重点企业正常生产和已建成项目达产达标。加强中小企业培育，加大对入规企业扶持力度，新增入规企业9户。加快水电冶金产业融合发展。支持企业加强技术研发中心建设，围绕节能节水和资源综合利用加强技术改造，促进循环经济发展。三是加快开发区建设步伐。围绕打造国家级经济技术开发区，抓紧修订完善陇南市经济技术开发区发展规划，抓好各县区开发区规划编制，完善开发区管理体制和产业布局，强化土地、政策保障，加快建设开发区融资平台，进一步推进开发区基础设施建设，全方位开展招商引资工作，在企业入园和项目落地上取得突破。四是以小微企业发展推动非公经济集中突破。进一步推动登记审批制度改革，切实降低准入门槛，鼓励民营企业进入基础设施、市政公用事业、矿产资源开发、社会事业、文化旅游产业等领域。依托开发区和电子商务产业园，建设非公企业孵化基地。大力发展个体工商户、私营企业、农民合作社等非公经济组织，引导个体工商户转型升级为私营企业。全面落实扶持政策，进一步强化对小微企业财税、金融、人才、用地等方面的支持，为小微企业发展创造良好环境。

　　三、以小城镇综合改革为重点，加快推进城乡一体化建设

　　着眼构建覆盖全市城乡的新型城镇化体系，统筹抓好市区、县城、建制镇、新农村建设。一是发挥规划引领作用。完善新型城镇化总体发展规划，明晰中心城市、县城、小城镇和新型农村社区建设定位和发展目标，强化规划引导功能，完善城镇路网、园林绿化、公共服务设施等专项规划，充分彰显陇南地域特点和文化特色，提升城镇建设文化内涵。二是深入推进小城镇综合改革试点。抓好礼县、康县及成县小川镇、宕昌县哈达铺镇省级新型城镇化试点，进一步加快9个市级小城镇综合改革试点镇基础设施、公共服务和产业发展项目建设，抓好土地管理和使用、投融资体制、户籍制度、政府职能转变等综合配套改革，力争今年有实质性突破，产生示范带动作用。三是加快重点市政项目建设。大力推进市区和八县县城建设，抓好市区东江新区开发、吉石坝新区建设和旧城区改造提升，加快实施火车站站前广场、滨江体育场、阶州文化城等重点项目。实施市区长江大道东西延伸二期工程。迁建东江污水处理厂，新建吉石坝污水处理厂，提高市区污水处理能力。抓好全市境内兰渝铁路沿线站场建设，为兰渝铁路年内在陇南通车提供条件。抓好八县一区城区水电气暖、公共交通、垃圾污水处理等市政基础设施项目。不断提高城镇和中心村公用服务设施网络化程度。四是着力提高城乡综合管理水平。突破城乡二元结构，创新城乡管理理念，将社会管理重点向农村延伸。以创建文明县城、卫生城镇和美丽乡村建设为载体，下功夫整治城乡环境卫生，提高城乡建设与管理水平。在旧城改造和棚户区改造中，统筹解决好小街小巷、背街背巷硬化亮化问题。

　　四、加快生态文明建设，努力建设长江上游生态安全屏障

　　树立绿色发展、循环发展、低碳发展的理念，坚持保护与开发并重，加快生态建设，着力构建生态安全屏障。一是大力实施生态修复和治理工程。坚持山水林田一体推进，全力实施“两江一水”区域综合治理、水生态文明城市建设试点等重大项目。抢抓机遇，实施好17万亩新一轮退耕还林工程，采取集中连片推进的方式，整山系、整流域发展经济林，加快建设“一城两带”经济林基地。抓好天然林保护、长防林工程、公益林补偿、文明长廊建设等重大生态工程，在生态脆弱区域实行封山禁牧。完成造林30万亩以上，建设油橄榄绿色通道400公里。抓好森林、湖泊、湿地等生态资源和自然保护区、风景名胜区保护管理工作。深入实施小流域综合治理、水土流失治理、梯田建设等工程。积极申报裕河国家级自然保护区，加快康县梅园沟、文县黄林沟国家湿地公园项目建设。二是加强环境污染治理。全面实施新《环保法》，持续推进污染减排和重金属污染治理，加大水污染、大气污染治理力度，严格控制排污总量，积极淘汰落后产能。深入推进农村环境综合整治，抓好美丽乡村示范村和环境整洁村建设，加强农业面源污染和规模养殖污染治理，持续开展废旧农膜回收利用，加快农村清洁能源建设。抓好两当县国家级生态文明建设示范区创建工作。三是强化防灾减灾工作。加强监测预报、灾害预防和应急救援体系建设，提高灾害防御能力。抓好江河干支流、中小河流治理，加快实施堤防、病险水库除险加固等项目，新建堤防150公里。加强防汛抗旱减灾和地质灾害防治工作，开展暴雨、山洪灾害应急抢险等实战演练。强化城乡建设工程抗震设防管理，加强防震减灾知识宣传和专业队伍建设。完善自然灾害监测预警指挥系统，建成新一代天气雷达系统。

　　五、大力推进项目建设，不断增强经济社会发展后劲

　　抢抓国家加大西部地区基础设施、社会事业、民生保障类建设投入等重大机遇，积极向国家、省上汇报衔接，争取实施一批重点项目。加强重大项目协调服务，力争兰渝铁路陇南至广元段年底通车运营，确保十天高速陇南段建成投用，渭武高速全线开工建设，徽两高速上半年开工建设，成州机场完成年度投资计划，全市高速公路里程达到380公里。争取武九高速年内完成前期工作，力争早日开工建设。做好天武铁路、阳九铁路前期工作。积极争取地方公路与国家公路连接线项目，抓好国省干线改造升级和出口路、联网路建设。确保礼武公路建成通车。加快建设礼县至康县、康县至武都、麻子川至角弓、东峪口至青龙桥公路，抓好徽县至略阳、康县至阳平关、裕河至青木川省级联网路建设。完成全市旅游路规划，争取把我市列为全省旅游路建设先行市。高标准建设重点景区和周边乡村公路。持续推进县乡公路建设和通村道路硬化工程，为2016年实现建制村通畅目标打好基础。加强公路养护管理工作，确保公路建设项目发挥最大效益。抓好民生水利项目，加快实施安全饮水工程，抓好5万亩油橄榄高效节水工程、小水电增效扩容和以电代燃料项目。加强电网建设，力争徽县330千伏变电站年内开工，建成330千伏天-晒-都二回线。加快通信基础设施建设，实施“宽带中国、光网城市”工程，实现八县一区城区全光化和50个乡镇光纤到户，行政村宽带覆盖率由60%提升至80%。加快智慧城市建设，实施智慧城市无线网络全覆盖项目。认真做好“十三五”规划编制工作。

　　六、加快发展电子商务，提升三产发展水平

　　坚持以电子商务、企业培育和市场体系建设为重点，推动现代服务业与传统服务业协调发展。一是推进电子商务再上新台阶。以促农增收为目标，以创建全国电商扶贫示范区为重点，不断放大农产品电子商务“陇南模式”带动效应。抓好电子商务产业孵化园建设，加快网络宽带和物流快递服务体系建设，加大对网销产品生产企业和供货商的扶持力度，促进网商、供货商、物流商形成完整产业链条，大力扶持和培育一批电子商务示范企业和示范网店，争取“陇南电子商务职业技术学院”早日挂牌，抓好成县、武都阿里巴巴农村电商“千县万村”项目，让电子商务在带动就业创业、增加群众收入、扩大消费等方面发挥更大作用。二是加快发展现代服务业。积极扶持传统服务业发展，制定完善优惠政策，在业务审批、准入等方面降低门槛，营造良好环境。加快发展技术研发、信息服务、文化创意、养老、保险、体育等现代服务业。积极培育消费市场，发挥消费对经济增长的拉动作用。三是加强商贸流通和市场体系建设。抢抓交通条件改善的重大机遇，积极培育连锁商贸企业，加强商贸综合体建设。抓好陇南特色农产品市场建设，抓好武都吉石坝、宕昌哈达铺和成县航空港物流园区建设，加快铁路沿线物流园区建设。抢抓国家“一带一路”、丝绸之路经济带甘肃黄金段建设和陇南向南开放的历史机遇，积极谋划运用好长江经济带、成渝经济圈，做实与我市相邻省市合作共赢这篇大文章，加快建设甘肃向南开放的桥头堡。

　　七、大力发展文化旅游产业，推动特色文化大市建设

　　把文化旅游产业作为适应新常态的重要抓手，推进文化旅游产业深度融合发展。围绕“6551”文化产业体系和“1313”特色文化大市建设，抓好省列20个大景区之一的宕昌官鹅沟景区、大堡子山国家考古遗址公园和长道三国古城建设。加大文物保护投入，抓好古建筑、古遗址保护工作，恢复性开发阶州文化城。加大传统古村落项目申报力度，抓好哈达铺镇、碧口镇、哈南村、杨店村等历史文化名镇名村和传统村落保护开发工作。加强白马文化、氐羌文化等民俗文化和非物质文化遗产保护传承，积极申报省级和国家级非物质文化遗产项目，做好“乞巧节”和“蜀道”、“北茶马古道”申报世界文化遗产工作。加强文化产业招商引资工作，实施好24个已签约项目。抓好乞巧文化宣传工作，不断提升中国陇南乞巧女儿节的影响力。办好第一届中国(陇南文县)白马人民俗文化旅游节。加大文化企业扶持力度，加强文化人才培养，打造文化精品工程，进一步推动文化艺术事业繁荣发展。实现文化产业增加值增长38.5%。开好全市旅游发展大会，破解我市旅游发展中的瓶颈与障碍。理顺旅游景区管理体制，完善旅游发展投融资机制，成立陇南市旅游发展公司。加大A级景区创建力度，做好宕昌官鹅沟创建国家5A级景区工作。抓好康县花桥村乡村旅游示范村建设，精心打造75个全国乡村旅游扶贫重点村，确保先行先试的旅游建设项目可复制、可推广，推动全市乡村旅游示范建设。规划实施好五阳路亚热带生态文化旅游圈建设。抓好原生态中医药养生保健旅游创新区建设，启动武都姚寨沟中医药养生试点工作。

　　八、加强财政金融工作，提高支撑发展能力

　　切实加强财源建设，挖掘增收潜力，加大征收力度，确保财政收入持续稳定增长。优化财政支出结构，严格落实厉行节约各项规定，把资金用在保民生、保重点上。深化“3+1”金融模式的探索，提高金融支撑能力。深入推进农村物权抵押贷款交易试点，抓好农村物权“常年确权”与抵押贷款交易平台建设，建立起功能完善的农村物权抵押贷款交易中心，解决“三农”融资问题。积极开展中小微企业融资“资金池”试点，解决中小微企业融资难、融资贵问题。加快政府投融资平台组建和规范工作，提高土地储备中心融资能力，按照国务院对政府融资举债的新要求，积极使用PPP、BOT、TOT等项目融资方式，吸引民间资本投入，解决重大项目融资问题。切实加大贷款清收工作力度，进一步优化地方信用环境。创新政府各类产业基金使用模式，增强增信能力，放大基金效应。加强银企合作，鼓励金融机构加大对“三农”和中小微企业的支持力度，推动落实好双联惠农贷款、妇女小额担保贷款和中药材、易地扶贫搬迁、规模养殖、设施蔬菜、电子商务等惠民信贷政策。加快金融服务体系建设，争取兰州银行陇南分行尽快成立。

　　九、推进社会事业发展，努力开创民生建设新局面

　　不断加大教育投入，合理配置教育资源，优化教育布局，推进全市义务教育均衡发展，确保徽县、成县义务教育均衡发展达到国家评估验收目标。深入实施农村义务教育薄弱学校改造工程，加快职业教育、学前教育和特殊教育发展，抓好陇南师专升本和陇南卫生职业技术学院创建工作，不断提高教育质量，努力办人民满意的教育。积极争取和实施省部科技计划项目，加快科技成果转化应用。加快标准化村卫生室建设，做好传染病防治、疾病防控、妇幼保健等公共卫生工作。全面推开县级公立医院综合改革，启动实施城乡居民大病保险，稳步推进分级诊疗和医师多点执业，深入开展新农合支付方式改革，提高基本公共卫生服务和城乡居民医保财政补助标准，加强社会救助“一门受理，协同办理”窗口建设工作，切实减轻群众就医负担。完善公共文化服务体系，抓好村级文化活动室、农家书屋、广播电视户户通、县级数字影院等文化基础设施建设，深入实施“乡村舞台”建设工程，加快市区新华书店建设，提高公共文化服务水平。实施好体育惠民工程，加快发展群众体育运动，提高竞技体育水平。落实就业扶持政策，实施好各类扶持高校毕业生就业民生实事项目，新增城镇就业2.8万人以上。加强社会保障体系建设，加大社会保险资金投入，全面推进机关事业单位养老保险制度改革，启动实施城乡基本医疗、生育保险市级统筹，完善城乡低保动态管理机制，落实好最低生活保障政策。加快推进保障性住房建设，发放好城镇困难家庭廉租住房补贴。全面落实党的民族宗教政策，加快民族乡村经济社会发展。加强民兵预备役建设和双拥共建工作，持续开展全民国防教育，重视发展老龄、残疾人、档案和红十字会事业，支持工会、妇联、共青团等群众团体工作，做好农村留守老人、留守儿童工作，维护好职工、妇女儿童和青少年合法权益。

　　坚持把改善民生作为政府要长期抓好的重点工程，集中力量办好10件民生实事：⑴完成150个扶贫整村推进；⑵硬化贫困村道路3000公里；⑶解决22万农村人口饮水安全问题；⑷建设“乡村舞台”600个；⑸完成棚户区改造7500套；⑹升级改造农村电网620公里；⑺实施易地扶贫搬迁1.8万人；⑻新建4G基站300个，实现农村无线网络全覆盖；⑼开展农业特色产业提质增效年活动，完成200万株核桃高接换优；⑽建成标准化村卫生室100所。

　　十、大力推进依法行政，不断提高社会治理能力

　　坚持在党的领导下、在法治轨道上开展工作，把法治思维和法治方式贯穿于政府工作全过程，坚持法定职责必须为，法无授权不可为，依法全面履行政府职责，自觉接受人大依法监督、政协民主监督等方方面面的监督。完善行政决策程序规定，健全依法决策机制，积极推行政府法律顾问制度，建立行政机关内部重大决策合法性审查机制，严格落实重大决策终身责任追究及责任倒查制度。深化行政执法体制改革，健全行政执法和刑事司法衔接机制。加强对行政权力的制约和监督，大力推进政务公开，重点推进财政预算、“三公”经费及公共资源配置、重大建设项目批准和实施、社会公益事业建设等领域的政府信息公开。围绕全面推进依法治市，健全宪法法律实施和监督制度，提高司法公信力。全面加强法律法规知识学习宣传培训。加强法制工作队伍建设，为建设法治陇南提供人才支撑。

　　深入推进社会治理创新。完善社会治理体制，全面推进社会依法治理。健全重大事项社会稳定风险评估机制和基层维稳应急处置机制，抓好群众信访和矛盾纠纷排查调处工作。加快社会管理信息系统建设。构建网格化社会治安防控体系，依法严厉打击违法犯罪，有效预防和减少各种不安全不稳定因素，着力提高人民群众安全感。大力培育发展公益性社会组织，探索政府购买公共服务措施，引导社会组织参与基层社会治理，提高基层治理水平。全面落实政府、部门、企业安全生产监管责任，加强重点行业领域安全监管，深入开展隐患排查治理和专项整治活动，确保人民群众生命财产安全。落实价格监管措施，确保市场价格保持基本稳定。抓好食品药品安全监管，发挥好乡镇监管机构职能作用，保障人民群众饮食用药安全。强化质量发展意识，严格落实质量工作目标责任制，加快推进全市经济提质增效升级。

　　十一、深入推进重点改革，全面加快对外开放

　　陇南经济社会发展已经站在了新的起点上，进入到深化改革、扩大开放、转型跨越的关键时期。我们将深入推进重点领域改革，不断增强发展活力。坚持把行政审批制度改革作为重要抓手，深入推进简政放权、放管结合，按照省政府的安排，再下放一批行政审批事项，进一步做好行政审批事项承接落实工作。建设市、县、乡三级政务服务网络。抓好政务服务中心的运营，使所有面向企业和社会的行政审批、行政收费和服务事项全部进驻政务大厅，建立一站式服务和审批制度，提高现场办结率。不断完善公共资源交易体系，进一步提高资源配置水平。突破体制机制制约，狠抓重点改革措施的落实，特别是在文化旅游、小城镇建设、水利改革、园区招商、社会治理等方面取得突破性进展。

　　我们将按照市委建设甘肃向南开放桥头堡的决策部署，大力推进向南开放，加快融入长江经济带、成渝经济圈，把接壤区域作为开放开发、交流合作的重点，积极探索合作机制，加强与川渝、陕南地区各个领域的务实合作，进一步抓好与汉中、广元、巴中、绵阳等邻近市州的交流合作，努力建设区域开放开发共同体。加强招商引资工作，进一步加大面向川渝地区的宣传推介力度，在企业引进、商贸物流和文化旅游合作、承接产业转移方面取得实质性进展。强化精准招商和以商招商，深化与友好城市交流合作，充分发挥商会的组织作用。提高签约项目落地率，实现招商引资到位资金385亿元以上。

　　各位代表，全面完成今年的各项工作任务，对政府自身建设提出了更高要求。我们将坚持不懈地加强政府自身建设，稳中求进，改革创新，抓住抓重，不断提高依法执政、科学执政、民主执政的能力和水平。全面落实好市委的总体工作部署，落实好经济工作目标责任，落实好人大及其常委会的各项决议、决定，认真办理人大代表建议和政协委员提案。对形成共识的大事要事，紧紧抓在手上，持之以恒地抓好落实。推动政府工作人员转变作风，努力解决群众关切的利益问题，认真落实中央八项规定、国务院“约法三章”和省委、市委一系列规定，全面落实党风廉政建设责任制，加强行政监察和审计监督，强化廉政风险防控，严肃查处各类违纪违法案件，树立清正廉洁的良好形象。

　　各位代表，新的征程已经开启,新的目标催人奋进。让我们在市委的坚强领导下，紧紧依靠全市人民，开拓创新，奋发进取，真抓实干，为建设幸福美好新陇南而努力奋斗!
